# Programming Chatbots Using Natural Language: Generating Cervical Spine MRI Impressions

**DOI:** 10.7759/cureus.69410

**Published:** 2024-09-14

**Authors:** Ramin Javan, Theodore Kim, Ahmed Abdelmonem, Ahmed Ismail, Farris Jaamour, Oleksiy Melnyk, Mary Heekin

**Affiliations:** 1 Department of Radiology, George Washington University School of Medicine and Health Sciences, Washington, D.C., USA; 2 Department of Research, California Institute of Behavioral Neurosciences & Psychology, Fairfield, USA; 3 Department of Radiology, Dartmouth-Hitchcock Medical Center, Hanover, USA

**Keywords:** ai chatbot, chatgpt, claude, computer programming, degenerative cervical spine disease, google bard, gpt-4, large language models (llm), llama, mri spine

## Abstract

Purpose: The utility of machine learning, specifically large language models (LLMs), in the medical field has gained considerable attention. However, there is a scarcity of studies that focus on the application of LLMs in generating custom subspecialty radiology impressions. The primary objective of this study is to evaluate and compare the performance of multiple LLMs in generating specialized, accurate, and clinically useful radiology impressions for degenerative cervical spine MRI reports.

Materials and methods: The study employed a comparative analysis of multiple LLMs, including OpenAI’s ChatGPT-3.5 and GPT-4 (OpenAI, San Francisco, CA), Antrhopic’s Claude 2 (Anthropic PBC, San Francisco, CA), Google’s Bard (Google Inc., Mountain View, CA), and Meta’s Llama 2 (Meta Platforms, Inc., Menlo Park, CA). This was performed during January-February 2024. These models were evaluated using a few-shot learning approach on a dataset consisting of 10 examples from 50 synthetically generated MRI reports. Performance metrics evaluated were diagnostic accuracy, stylistic accuracy, and redundancy.

Results: While Claude 2 maintained consistent high performance across 40 cases, GPT-4 required midway re-training to improve its declining scores. Both Claude 2 and GPT-4 demonstrated the ability to generate structured impressions, but Claude 2’s specialized summarization capabilities provided an edge in maintaining accuracy without continuous feedback. The other LLMs’ performance was subpar.

Conclusion: The findings of this study suggest that LLMs can be a valuable tool in automating the generation of radiology impressions. Claude 2, in particular, exhibited promising results, indicating its potential for clinical implementation. However, the study also points to the necessity for further research, especially in optimizing model performance and evaluating real-world applicability.

## Introduction

Large language models (LLMs) are a class of artificial intelligence (AI) systems that are trained on massive amounts of textual data to generate human-like text. They utilize deep learning neural network architectures, most commonly Transformer architectures, employing self-attention mechanisms to focus on relevant parts of long input sequences and recurrent connections to understand and influence context. this makes them well-suited for natural language tasks [1]. Unlike traditional statistical language models, LLMs like GPT-4 and Claude 2 are trained in a self-supervised manner on raw internet text data through a pre-training process, with subsequent fine-tuning paradigms [2]. Pre-training exposes the models to diverse language examples to build a strong general understanding. Fine-tuning, then, calibrates the pre-trained model using task-specific datasets and reinforcement learning from human feedback (RLHF) to optimize performance for specialized applications like language summarization, radiology report generation, etc. [3].

Multimodal models build upon standard LLMs by further incorporating non-textual data like images, audio, and video during training to support both text and image-based inputs and outputs [4]. They relate textual concepts to visual features using co-training on paired images and captions [5]. This expands their knowledge base and allows multimodal chatbots to not only process text prompts but also analyze images, including medical images. For example, models like GPT-4 Vision can process images as input and provide textual analysis [6].

Recent studies have evaluated LLMs for generating radiology reports and impressions. Sun et al. tested GPT-4's ability to write impressions for 50 chest radiographs from the National Institutes of Health chest radiography data set. They found that radiologist-generated impressions had higher coherence, comprehensiveness, factual consistency, and less medical harmfulness than GPT-4-generated impressions, mainly due to GPT-4 using unsupported statements, missing important information, and creating a certainty illusion [7]. Tang et al. evaluated the capabilities and limitations of LLMs like GPT-3.5 and ChatGPT in summarizing medical evidence reviews through automatic metrics and human evaluation. The results reveal that these models can struggle with generating factually consistent and comprehensive summaries without harmful misinformation, especially over longer textual contexts, determining that advanced fine-tuning and customizations were necessary for optimal and reliable performance [8]. Other groups have explored LLMs for data extraction from reports. Blüthgen proposed that large language models like ChatGPT can potentially be instructed to extract measurements, such as lung nodule sizes, from radiology reports; however, this capability has not yet been demonstrated [9]. Consequently, the potential for LLMs is to generate text and analyze radiologically relevant content, potentially comparing radiologic studies and reports over time or corroborating with relevant information in electronic medical records.

Beyond impressions and data extraction, researchers have assessed chatbots for additional applications relating to radiology reports. Adams et al. transformed 50 free text reports in four languages into structured templates using GPT-4 [10]. Bosbach et al. had ChatGPT generate full distal radius fracture reports after training on 3 examples, yielding generally high-quality reports [11]. In a clinical support role, LLMs have generated acceptable screening recommendations, triaging differentials, and protocol selections when guided by specific instructions [12-14]. Thus, despite limitations, these studies reveal LLMs’ potential versatility in radiology.

By providing immediate, accurate, and reliable impressions, LLMs can alleviate the workload for radiologists and improve communication with referring clinicians [15]. This is significant given the current circumstances where there is a shortage of radiologists [16]. However, the potential of chatbots in specialized radiology applications remains to be explored. To our knowledge, generating customized impressions across sub-specialties in radiology is yet to be tested. This study evaluates multiple chatbots for generating targeted degenerative cervical spine MRI impressions from structured reports using few-shot prompt engineering. 

## Materials and methods

We evaluated multiple chatbots for generating targeted degenerative cervical spine MRI impressions from structured reports. Models tested included Google’s Bard (Google Inc., Mountain View, CA), OpenAI’s ChatGPT-3.5 and GPT-4 (OpenAI, San Francisco, CA), Meta’s Llama 2 (Meta Platforms, Inc., Menlo Park, CA), and Anthropic’s Claude 2 (Anthropic PBC, San Francisco, CA). The evaluations were performed through few-shot prompt engineering, which involved providing an initial set of commands followed by multiple input-output example pairs.

**Figure 1:**
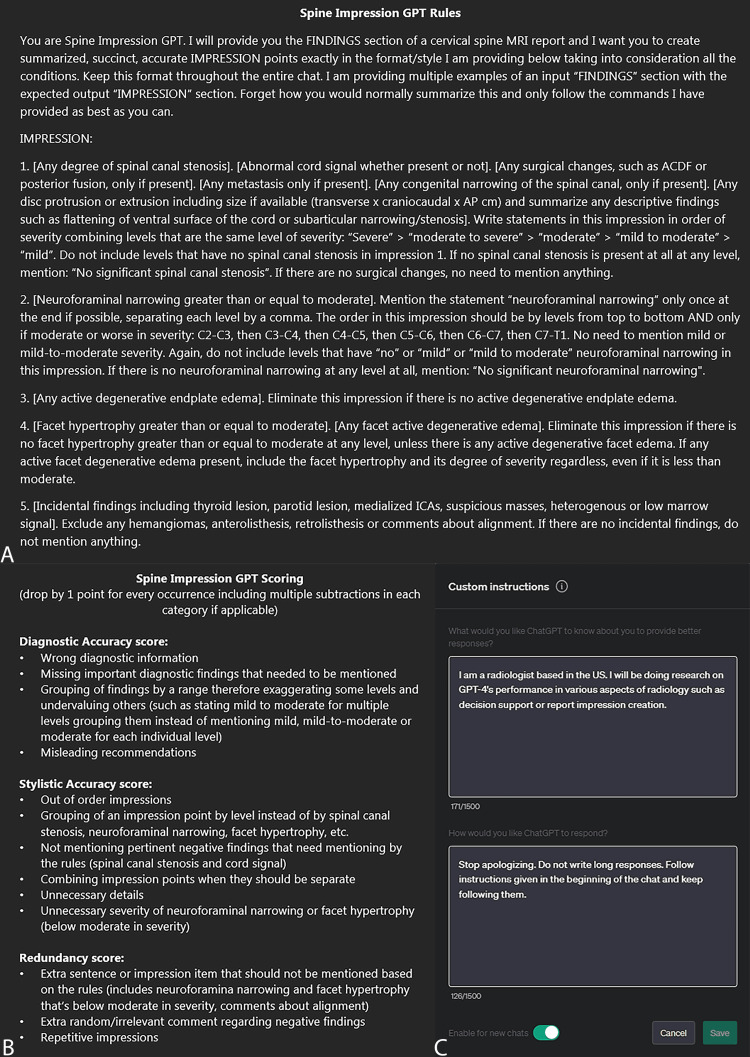
Spine impression GPT rules, impression scoring system, and ‘Custom Instructions’ settings A. The first section showcases the initial prompt (natural language programming), where all the rules are defined for the chatbot in creating the impressions based on the findings; B. The lower left section shows the impression scoring system used for acquiring the data; C. The lower right (1C) ‘Custom Instructions’ settings show specific additional broad guidance for GPT-4 to decrease verbosity and redundancy.

Specific instructions for generating custom degenerative cervical spine MRI impressions in a natural language format were first given to each chatbot (Figure 1A). This was followed by multiple interactions where 10 sample ‘Findings’ sections were inputted as examples, and the chatbots’ generated ‘Impression’ texts were then corrected through iterative feedback after each case during the training phase. Institutional review board review was not required as the reports provided to the chatbots were a total of 50 mock reports (again, 10 of which were for training purposes), representing varying degrees of complexity in degenerative findings. A scoring system for the generated impressions was used (Figure 1B) for grading the results of each chatbot. Specifically for GPT-4, custom instructions were broadly provided (Figure 1C) to reduce verbosity and redundancy. 

For Bard, ChatGPT-3.5, Llama 2, and set 1 of GPT-4 (without ‘Custom Instructions’), a set of 20 cases were tested. GPT-4’s set 2 evaluation comprised 40 cases in addition to filling in the ‘Custom Instructions’ setting, emphasizing brevity and professional language. Due to a drop in performance of GPT-4 midway through set 2 (set 2a), the model was re-trained through two prompts (the original rules and one example), and subsequently, the 20 remaining cases (set 2b) were tested. Lastly, Claude 2 was evaluated on the full set of 40 cases after training on 10 examples, without any re-training or additional feedback throughout the process. The generated impressions were scored by an experienced subspecialty neuroradiologist using a five-point Likert scale assessing three factors: diagnostic accuracy, stylistic accuracy, and redundancy.

Statistical analysis

To evaluate the inherent ability of models that showed a decline in performance, which may have been limited once the overall chat became too lengthy, the cases were arbitrarily divided into the first five and subsequent 15, allowing us to assess any changes in performance over time. The differences in performance metrics between the LLMs were analyzed using descriptive statistics to summarize accuracy, style, and redundancy ratings. For models like GPT-4 that required re-training, pre- and post-retraining scores were compared to evaluate the impact of iterative feedback on performance. The analysis also included case-by-case graphical comparisons to visualize the trends and deviations in model performance. No inferential statistical tests were performed, as the primary goal was to provide descriptive comparisons between models, and the acceptable GPT-4 performance was on such few initial cases before degradation (approximately six or seven), which did not provide adequate power for a statistically significant analysis. However, future studies may benefit from employing more robust statistical methods, especially when the performance of LLMs also becomes more consistent, spanning a larger number of sequential cases. In those cases, methods such as paired t-tests or ANOVA can be performed to evaluate the statistical significance of performance differences across models.

## Results

ChatGPT-3.5 and Llama 2 yielded unsatisfactory results from the start, not warranting further investigation. While Bard was able to follow instructions, it had a precipitous decline after approximately six or seven cases. Re-training of Bard was not pursued as the performance through 20 cases showed relatively mediocre levels of accuracy. GPT-4 was initially able to follow instructions but also had a precipitous decline after approximately six or seven cases when not receiving feedback. Providing information in the ‘Custom Instructions’ box of GPT-4 (Figure 1C) improved its performance. For GPT-4’s second set, the re-training process midway immediately raised performance to the original levels. Claude 2, however, not only showed high levels of accuracy from the start but maintained its performance throughout, as detailed below.

**Figure 2:**
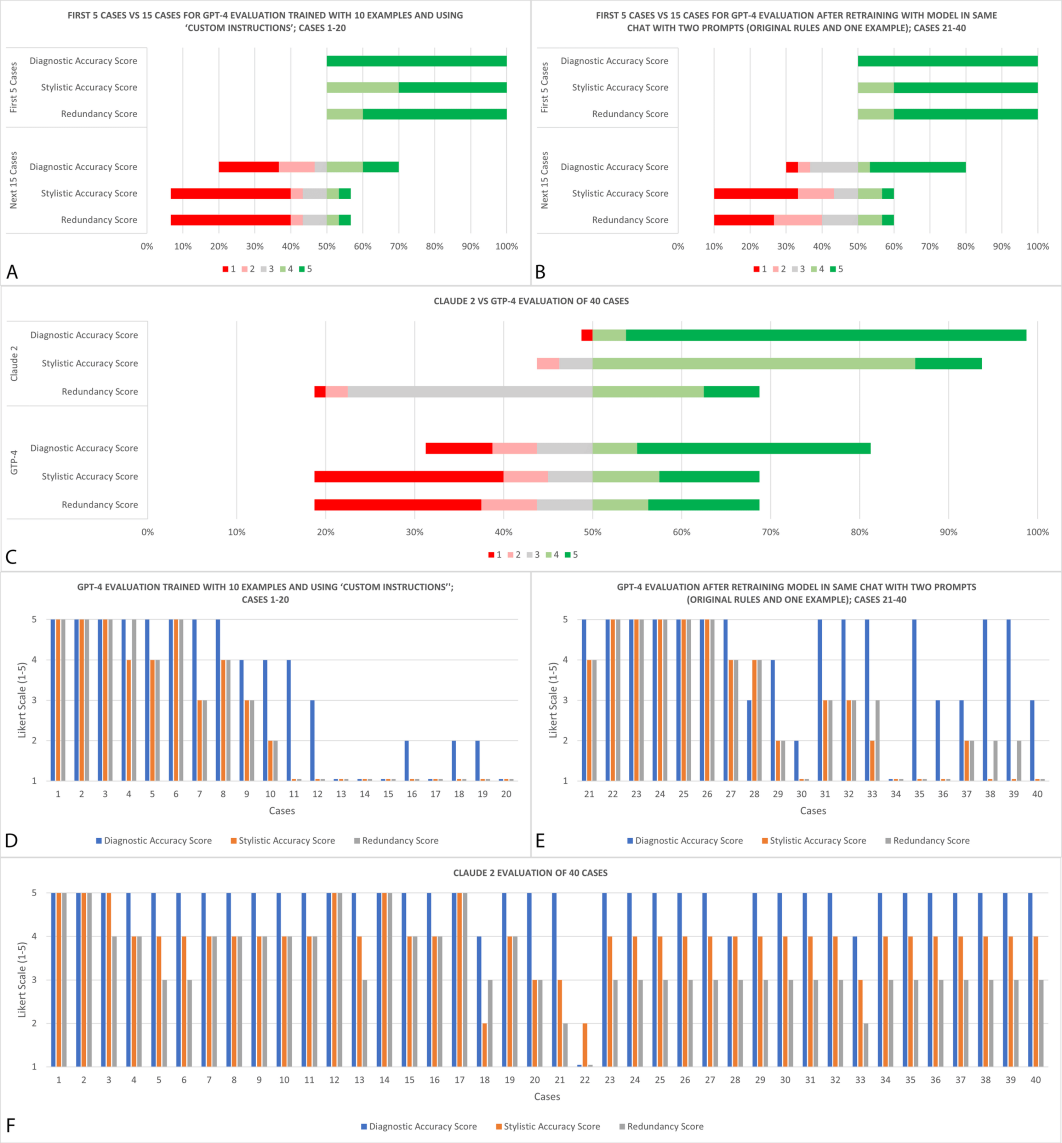
Performance of GPT-4 vs Claude 2 A. Diverging bar graphs of the Likert scale for the first five vs. subsequent 15 cases of GPT-4 as the first set of 20 cases; B. Diverging bar graphs of the Likert scale for the first five vs. subsequent 15 cases of GPT-4 of the second set of 20 cases after retraining with the original rules and one example as prompt; C. Comparison of diverging bar graphs of Likert scale between Claude 2 vs. GPT-4 for 40 cases; D. Case-by-case graph of Likert scores for GPT-4's performance in the first set of 20 cases; E. Case-by-case graph of Likert scores for GPT-4's performance in the second set of 20 cases after retraining with the original rules and one example as prompt; F. Case-by-case graph of Likert scores for Claude 2's performance on a set of 40 cases.

The diagnostic accuracy, stylistic accuracy, and redundancy scores (Figure 1B) in the Likert scale from one to five for GPT-4 and Claude 2 are presented as diverging bar chart graphs (Figures 2A-2C) and as case-by-case individually detailed (Figures 2D-2F) below. The mean Likert scale scores as percentages of the first five vs subsequent 15 cases for the chatbots were as follows: Bard (76% vs 33%, 68% vs 29%, 64% vs 29%), GPT-4 set 1 (92% vs 97%, 76% vs 33%, 56% vs 40%), GPT-4 set 2a (100% vs 55%, 92% vs 36%, 96% vs 36%), and GPT-4 set 2b (100% vs 79%, 96% vs 43%, 96% vs 47%).

**Figure 3:**
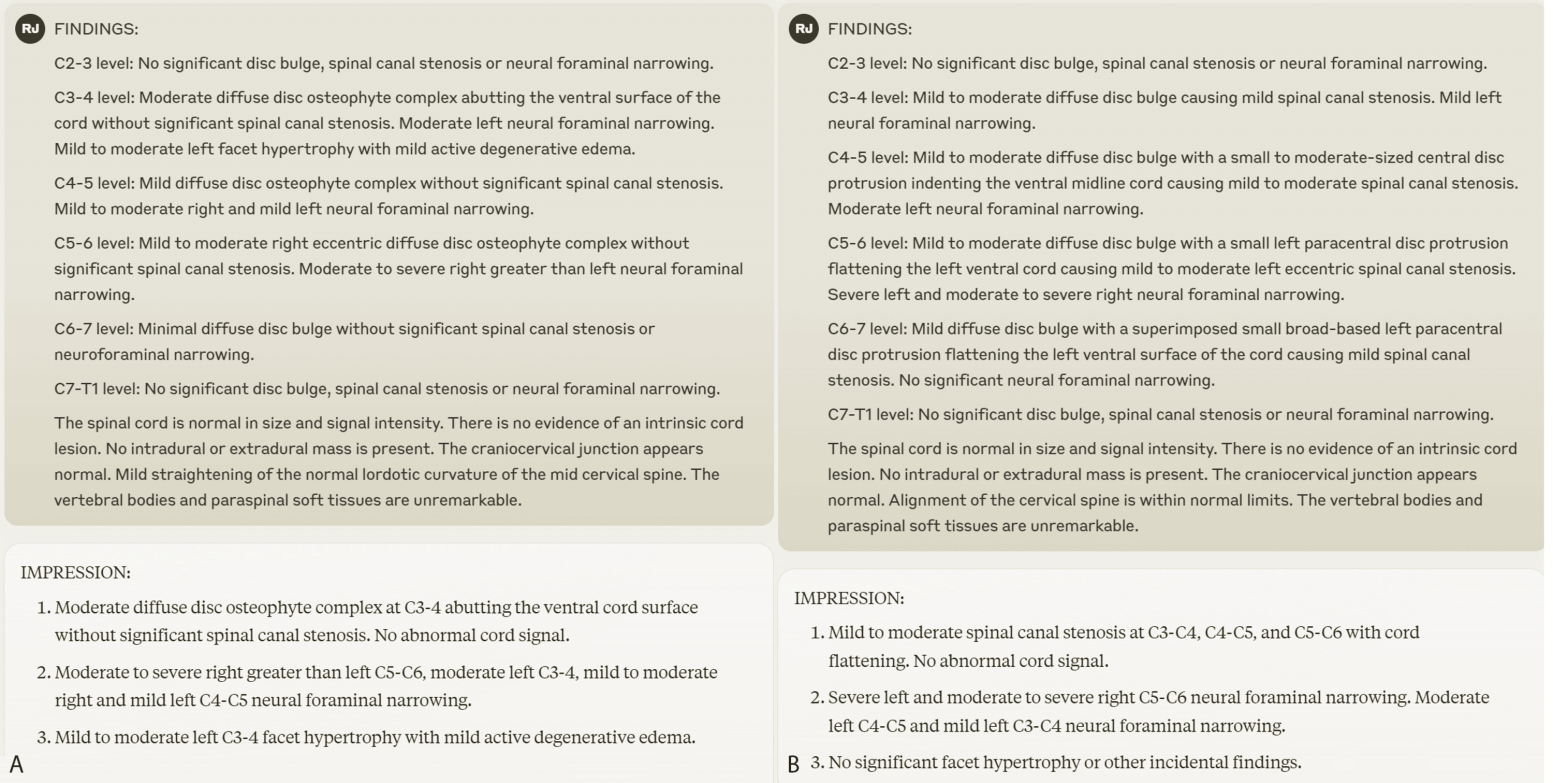
Example cases of performance of Claude 2 A, B: Example cases of performance of Claude 2 in generating degenerative cervical spine impressions from findings section.

Mean Likert scores for the 40 cases of GPT-4 set 2 (including mid-way re-training, Figures 2D-2E) vs. 40 cases of Claude 2 (without retraining, Figure 2F) were 75% vs. 97%, 51% vs. 80%, and 51% vs. 68%. The main factor in lowering stylistic and redundancy scores for Claude 2 was related to the repetition of the same negative impressions that were not required in the initial rules. Two example cases for Claude 2 are provided (Figures 3A-3B).

## Discussion

Chatbots are now widely available to the general public through multiple options and user-friendly interfaces. Our findings indicate that Claude 2 and GPT-4 can easily be programmed to generate structured impressions using natural language and formatting instructions. This opens the door to significant possibilities for any user without programming experience or coding background. However, some important limitations should be considered, including the drop in performance of GPT-4 over prolonged conversations, lack of testing on a large number of real patient reports, and evaluation by only a single observer/reader in this study. 

While Claude 2 was able to maintain performance throughout testing, the tested iteration of GPT-4 (Jan 10, 2024 version) experienced a concerning decline. There are several potential reasons for this drop in accuracy. First, as the conversation progresses, the initial information likely becomes less important to the model. Second, the growing number of tokens exchanged between the user interface chat environment can increase computing needs and costs substantially, which may therefore be deliberately limited by OpenAI to control for cost. Third, “AI drift” may occur, where newer versions of an AI tool degrade as more constraints are added for alignment purposes [17].

Fortunately, prompt engineering offers a promising approach to maximize chatbots' specialized capabilities and to obtain narrow, desirable results through carefully crafted inputs [3]. Of note, one of the specialized capabilities of LLMs is summarization, which is a subfield of natural language processing [8]. Anthropic’s Claude 2 is a freely available, lesser-known LLM compared to GPT-4, but has an especially powerful summarization capability and a significantly higher limit for total word input [18]. Ultimately, to achieve reliable results without the need for continuous feedback, both Claude 2 and GPT-4 will likely require advanced fine-tuning techniques (training the core engine of an LLM through Python code with dozens of input/output pair examples) and/or AutoGPT through the use of AI agents [19,20].

Furthermore, multimodal models like GPT-4 Vision introduce new, exciting possibilities by processing visual data. Early testing revealed it could caption ultrasound images of the thyroid and generate 3D renderings from MRI slice descriptions [6]. However, significant challenges exist. Studies found it incorrectly labeled fundus photographs and created nonsensical graphics from prompts, indicating the need for specialty training [21]. Still, capabilities like generating simulated medical images could greatly benefit research. Artificial intelligence-powered image generators such as Midjourney and Dall-E 3 also add another dimension to potential applications in radiology that remain to be explored [22]. Integrating computer vision alongside robust language proficiency paves the way for AI radiology assistants, as well as the addition of custom GPTs, allowing for the inclusion of additional and custom knowledge bases and any combination of skills [23].

Yet, despite the excitement over LLMs' potential, appreciating their limitations is prudent. A major concern is artificial hallucinations, where chatbots confidently generate false information unrelated to the prompt with high confidence [24]. As models rely exclusively on pattern recognition from their training data, they lack true comprehension and can fabricate content if the requests are too ambiguous or fall outside their knowledge domain [25]. Another issue is concept drift, as mentioned previously, where the response quality gradually declines over prolonged conversations as earlier context gets neglected [26]. Failures can also arise when instructions are too vague or models are applied beyond their intended use cases.

In addition, challenges exist around privacy and bias. Patient privacy is another serious consideration, as large volumes of protected health data would likely be needed for optimal medical training. Potential solutions could include federated learning, as it keeps data decentralized during model development, and the use of local language models and open-source LLMs [27]. Finally, biases encoded in the training data, like gender or racial skews, risk propagating through AI systems and affecting patient care [28]. Ongoing initiatives aim to address these limitations through techniques like alignment and accountability [29].

Last, one of the limitations of research in the field of LLMs, including our study, is the constant development of newer iterations and more advanced models. Subtle changes to model architecture between versions can significantly impact performance, complicating efforts to compare studies that use different release versions of chatbots. For example, at the time of this submission (August 2024), the newest version of Claude is the 3.5 Sonnet, Meta’s Llama is the 3.1 version, Bard is now called Gemini, and ChatGPT’s latest iteration is GPT-4o. A repeat of this same study would now likely yield differing and perhaps better results than our current study.

However, our main goal was to demonstrate the programmability of these tools using natural language and the ability for any individuals to do the same. Future work should consider extending the scope of the study to include thoracic and lumbar spine MRI, as well as CT scans, to develop a more comprehensive tool for generating impressions across a range of degenerative spinal conditions. Subsequent research could also explore the capabilities of these models in addressing various types of spinal conditions beyond degenerative issues, such as multiple sclerosis. Last, assessing the real-world impact of these models through their implementation in actual clinical cases could provide valuable insights into their potential to enhance efficiency, coherence, and consistency, and to improve referring physician satisfaction [30]. The productivity gains could be quantified by measuring the time saved per report or across multiple reports, and this data could then be extrapolated to estimate the impact over a day, month, or year, shedding light on potential resource savings, mental energy conservation, and financial benefits.

## Conclusions

Advanced LLMs demonstrate the potential to transform radiology but require responsible and evidence-based implementation. This study found that Claude 2 and GPT-4 could generate structured cervical spine MRI impressions when provided explicit instructions, but Claude 2 better-maintained accuracy without needing continuous feedback. However, GPT-4 declined in performance over prolonged use, indicating specialized tuning through reinforcement learning is necessary for reliable results. While excitement surrounds LLMs in radiology, carefully evaluating their limitations around hallucinations, drift, privacy, bias, and stability remains imperative before clinical adoption. As these AI technologies continue rapidly evolving, it is essential that users appreciate their capabilities and constraints to harness them most effectively.
